# Perinatal Outcomes and Factors Associated with Ethnic Group in cases of Preterm Birth: the Multicenter Study on Preterm Birth in Brazil

**DOI:** 10.1055/s-0041-1739492

**Published:** 2021-12-06

**Authors:** Karayna Gil Fernandes, Renato Teixeira Souza, Renato Passini, Ricardo Porto Tedesco, José Guilherme Cecatti

**Affiliations:** 1 Department of Obstetrics and Gynecology, Faculdade de Ciências Médicas, Universidade Estaudual de Campinas, Campinas, SP, Brazil; 2 Department of Obstetrics and Gynecology, Faculdade de Medicina de Jundiaí, Jundiaí, SP, Brazil

**Keywords:** preterm birth, ethnic group, maternal outcomes, perinatal outcomes, parto prematuro, grupo étnico, resultados maternos, resultados perinatais

## Abstract

**Objective** To investigate the characteristics of women who had preterm birth (PTB) and related outcomes according to ethnicity.

**Methods** A secondary analysis of a multicenter cross-sectional study conducted in Brazil. Women who had PTB were classified by self-report as white and non-white. Clinical, pregnancy, and maternal data were collected through postpartum interviews and reviews of medical charts. The sociodemographic, obstetric and clinical characteristics of the women, as well as the mode of delivery and the neonatal outcomes among different ethnic groups were compared through a bivariate analysis.

**Results** Of the 4,150 women who had PTB, 2,317 (55.8%) were non-white, who were more likely: to be younger than 19 years of age (prevalence ratio [PR]: 1.05; 95% confidence interval [95%CI]: 1.01–1.09); to be without a partner; to live on low income; to have lower levels of schooling; to have ≥ 2 children; to perform strenuous work; to be from the Northeastern region of Brazil rather than the from Southern region; to have a history of ≥ 3 deliveries; to have an interpregnancy interval < 12 months; to have pregnancy complications such as abortion, PTB, preterm premature rupture of membranes (pPROM), and low birth weight; to initiate antenatal care (ANC) visits in the second or third trimesters; to have have an inadequate number of ANC visits; to be under continuous overexertion; to smoke in the first and second or third trimesters; and to have anemia and gestational hypertension. The maternal and neonatal outcomes did not differ between the groups, except for the higher rate of low birth weight (73.7% versus 69.0%) in infants born to non-white women, and the higher rate of seizures (4.05% versus 6.29%) in infants born to white women.

**Conclusion** Unfavorable conditions were more common in non-whites than in whites. Proper policies are required to decrease inequalities, especially in the context of prematurity, when women and their neonates have specific needs.

## Resumo

**Objetivo** Investigar as características das mulheres com parto pré-termo e os respectivos resultados de acordo com a etnia.

**Métodos** Uma análise secundária de um estudo de corte transversal multicêntrico no Brasil. Mulheres com parto pré-termo foram classificadas por autodefinição como brancas ou não brancas. Dados maternos, clínicos, e da gestação foram coletados por entrevista pós-parto e revisão de prontuários. As características sociodemográficas, obstétricas e clínicas das mulheres, o tipo de parto, e os resultados neonatais dos grupos étnicos foram comparados por análise bivariada.

**Resultados** Das 4.150 mulheres que tiveram parto pré-termo, 2.317 (55,8%) eram não brancas, que com mais frequência: eram menores de 19 anos de idade (razão de prevalência [RP]: 1,05; intervalo de confiança de 95% [IC95%]: 1,01–1,09); não tinham parceiro; eram de baixa renda; tinham baixa escolaridade; tinham ≥ 2 filhos; realizavam trabalho extenuante; provinham mais do Nordeste do que do Sul; tinham histórico de ≥ 3 partos; tinham intervalo interpartal < 12 meses; e tiveram complicações gestacionais como aborto, parto pré-termo, rotura prematura de membranas pré-termo (RPM-PT) e baixo peso ao nascimento; iniciaram as consultas de pré-natal no segundo ou terceiro trimestres; compareceram a um número inadequado de consultas; viviam sob contínua exaustão; fumaram no primeiro e segundo ou terceiro trimestres; e tiveram anemia e hipertensão gestacional. Os resultados maternos e neonatais não diferiram entre os grupos, exceto pela maior taxa de baixo peso ao nascimento (73,7% *versus* 69,0%) entre as crianças das mulheres não brancas, e e a maior taxa de convulsões (4,05% *versus* 6,29%) entre as das brancas.

**Conclusão** Condições desfavoráveis foram mais comuns entre não brancas do que entre brancas. Políticas apropriadas são necessárias para diminuir as diferenças, especialmente no contexto da prematuridade, quando mulheres e seus neonatos têm necessidades específicas.

## Introduction

Preterm birth (PTB) is a public health problem that may affect different strata of the population in an unequal manner. In general, PTBs affect 1 in every 8 infants born in Brazil, and is the main cause of neonatal morbidity and mortality. 1 2 3 Preterm birth has a huge impact on all those involved (the individual, the family, or the community). 3

It is widely known that maternal age, race/ethnicity, smoking, marital status, and socioeconomic level are factors related to a higher probability of having PTB. 1 4 Racial/ethnic inequality is related to an increased risk of PTB in black women, although the determinants of ethnic disparity in PTBs are still unknown, particularly in extreme PTBs. 5 Studies 6 have shown higher PTB rates among black women, which may be justified by social disparities. Nevertheless, this association remains obscure. Some studies 7 have shown that, even after adjusting for this potential bias (social disparity), PTB rates continue to be higher among black women. In 2018, a systematic review 8 showed that PTB is 1.5 times more common among black women than among non black women. The study 8 also concluded that there is a paucity of studies evaluating the ethnic aspects involved in this risk relation, and the assessment of the results from the literature search conducted by the authors suggested a publication bias.

Maternal stress before and during pregnancy and genital tract infection also increase the risk of PTB, 9 10 in alignment with another study 11 that showed that black infants are more likely to be born preterm in a metropolitan area with racial segregation than an infant born to a black woman living in a non-segregated metropolitan area, demonstrating that the environment where the woman lives exerts an influence on pregnancy outcomes. 9 10 11

Factors related to PTBs have not yet been completely elucidated. Furthermore, the distribution of these factors by race among the population is still incompletely explored. Advances in the identification of populations at risk of PTB and the recognition of the burden of consequences of PTB are of the utmost importance for the development of public policies intended to minimize the impact of this public health problem. The aim of the present study is to investigate the ethnic differentials in the characterization and determination of PTBs and their respective maternal and neonatal outcomes, according to a multicenter study conducted in Brazil.

## Methods

The present study is a secondary analysis of the Multicenter Study on Preterm Birth in Brazil (Estudo Multicêntrico de Investigação de Prematuridade no Brasil, EMIP, in Portuguese), a cross-sectional study in which the authors conducted a prospective surveillance of all PTBs occurring from April 2011 to July 2012 in 20 referral hospitals distributed throughout the 3 most populated regions of Brazil (Southern, Southeastern, and Northeastern). 3 12 13 The current analytical approach is to evaluate an association between ethnicity (defined by skin color) as an exposure factor and preterm deliveries. Two groups were considered: whites and non-whites. Maternal and perinatal outcomes were compared in both groups. In addition, an association between other maternal and pregnancy characteristics and PTB among non-white women was also investigated.

The methodological details of this study have already been described in other EMIP publications. 3 12 13 In brief, the participating centers conducted a prospective surveillance of 33.740 deliveries during the study period, including all pregnant women admitted due to PTB during the study period (less than 37 weeks of gestation), irrespective of the cause. The women were informed about the study. Data collection began after these women agreed to participate in the study and signed the consent form. Maternal and newborn data were collected in the postpartum period through a structured questionnaire applied by a duly trained research assistant. Information was collected through a review of the medical charts and in-person interviews with the woman prior to hospital discharge. Neonatal data were collected until hospital discharge or until 60 days postpartum.

For data standardization, in-person training was carried out to explain each step of data collection and data insertion into the specific platform of the study. For this procedure, an interviewer's manual was specially designed for the study, including all categories possible for each variable in the form, in addition to the procedures to which these women had been submitted. 13 The data obtained were later typed into an electronic form (in the OpenClinica 3.0 platform) developed specifically for the study and available on the web page of the coordinating center of the study (Universidade Estadual de Campinas, UNICAMP, in Portuguese). The calculation of the sample size considered that the prevalence ratio (PR) of PTB in Brazil was of 6.5% in 2006. As a result, each subgroup required at least 1.054 women. The subgroup of spontaneous PTB contained patients with preterm premature rupture of membranes (pPROM) and those who started preterm labor spontaneously. 3

For the current analysis, exposure variables were determined according to self-reported ethnicity. Women were then divided into a white and a non-white groups. To address whether maternal characteristics significantly vary according to ethnicity, maternal characteristics were divided into sociodemographic, obstetric history, and clinical care and antenatal care (ANC) characteristics. The sociodemographic variables included region of the country, maternal age, marital status, schooling, monthly income, number of children under 5 years of age, paid work during pregnancy, perceived strenuous work, and daily workload. Obstetric history (only for women with a previous pregnancy) included parity, and previous cesarian section, abortion, interpregnancy interval, PTB, pPROM and low birth weight. Finally, the clinical care and ANC characteristics were trimester when ANC visits began, adequate number of ANC visits, weight gain during pregnancy according to the first recorded weight before 20 weeks and the last recorded weight (the respective week of gestation was also recorded), perceived physical effort during pregnancy, smoking, urinary tract infection (including asymptomatic bacteriuria), vaginal bleeding, anemia (based on self-reports or medical records), chronic hypertension, diabetes (both preexisting or gestational diabetes), gestational hypertension, preeclampsia/eclampsia/hemolysis, elevated liver enzymes, and low platelet count (HELLP) syndrome, fetal malformation, fetal growth restriction, and multiple pregnancy.

A bivariate analysis was performed to determine the higher prevalence of different characteristics according to ethnicity using the PRs and the respective 95% confidence intervals (95%CIs). Maternal and neonatal outcomes were compared using proportions (expressed as percentages) and the Chi-squared test. The significance level adopted was of 5%. The Statistical Analysis System (SAS, SAS Institute, Cary, North Carolina, US) software for Windows, version 9.4, was used.

The study followed all international and national ethical guidelines for human research. All participants received information and instructions about the study. A consent form was read to dispel any doubts, which was signed after each woman agreed to participate in the study. The women were reassured that their identity would remain confidential, regardless of their participation in the study. The research was conducted in full compliance with the Declaration of Helsinki, and it was approved by the review board of the coordinating center and the Brazilian National Commission on Ethics in Research (Comissão Nacional de Ética em Pesquisa, Conep, in Portuguese) before the study began (Letter of approval 704/2009). Each participating center subsequently obtained approval from their local ethics committees before the study began.

## Results

During the study period, surveillance of 33,740 deliveries was conducted, and 4,150 women who had PTB (12.3%) were identified and included in the EMIP study. Of the total number of women who had PTB, 1,833 (44.2%) were self-reported whites. and 2,317 (55.8%) were self-reported non-whites. Although the proportion of non-white women was statistically lower in the PTB group than in the term group, 3 maternal outcomes such as the subtype of PTB, onset of labor and mode of delivery did not differ between the groups ( Table 1 ).

**Table 1:** Maternal outcomes of women who had preterm birth according to ethnic group

Characteristics	Non-white women	White women	Total	*p-value*
	n (%)	n (%)		
Preterm birth				
Spontaneous	839 (36.2)	652 (35.6)	1,491	0.6932
Therapeutic or elective	793 (34.2)	675 (36.8)	1,468	0.0826
Preterm premature rupture of membranes	685 (29.6)	506 (27.6)	1,191	0.1767
Onset of labor				
Spontaneous	1,265 (54.6)	952 (51.9)	2,217	0.1080
Elective cesarian	752 (32.5)	607 (33.1)	1,359	
Induced labor	300 (12.9)	274 (14.9)	574	
Mode of delivery ^a^				
Vaginal	1,078 (47.0)	803 (44.6)	1,881	0.3011
Cesarean	1,188 (51.8)	976 (54.2)	2,164	
Forceps/Vacuum	28 (1.2)	23 (1.3)	51	
Total	2,317 (55.8)	1833 (44.2%)	4,150	

Note: ^a^ Missing data for 54 cases.

It was more likely that non-white women were: aged ≤ 19 years (PR: 1.05; 95%CI: 1.01–1.09); did not have a partner (PR: 1.09; 95%CI: 1.02–1.16); had a low monthly income (PR: 1.31; 95%CI: 1.24–1.39); had a low level of schooling (< 8 years; PR: 1.35; 95%CI: 1.19–1.53); had ≥ 2 children (PR: 1.19; 95%CI: 1.07–1.33); and performed strenuous work (PR: 1.16; 95%CI: 1.05–1.27) compared to white women ( Table 2 ). Non-white women were more likely to be from the Northeasteran region and less likely to be from the Southern region of Brazil (2.7-fold and 0.54-fold respectively) ( Table 2 ).

**Table 2:** Maternal sociodemographics

Sociodemographics	Non-white women		White women		Prevalence ratio (95% confidence interval)
	n	(%)	n	(%)	
Region of Brazil					
Southeastern	1,162	(50.2)	1,127	(61.5)	1
Northeastern	1,011	(43.6)	330	(18.0)	**2.72 (2.36–3.14)**
Southern	144	(6.2)	376	(20.5)	**0.54 (0.47–0.63)**
Maternal age (years) ^a^					
≤ 19	506	(21.9)	358	(19.5)	**1.05 (1.01–1.09)**
20-34	1,502	(64.9)	1,178	(64.3)	1
≥ 35	308	(13.3)	297	(16.2)	1.04 (0.99–1.09)
Marital status					
Without partner	570	(24.6)	385	(21.0)	**1.09 (1.02–1.16)**
With partner	1,747	(75.4)	1,448	(79.0)	1
Schooling (years) ^b^					
< 8	1,005	(44.1)	636	(35.2)	**1.35 (1.19–1.53)**
8-12	1,118	(49.1)	987	(54.6)	**1.17 (1.04–1.33)**
> 12	154	(6.8)	186	(10.3)	1
Monthly income ^c^					
> US$ 500	1,612	(76.2)	1,454	(86.6)	1
≤ US$ 500	504	(23.8)	225	(13.4)	**1.31 (1.24–1.39)**
Children under the age of 5 ^d^					
No	1,649	(71.2)	1,388	(75.8)	1
1	546	(23.6)	377	(20.6)	**1.09 (1.01–1.16)**
≥ 2	121	(5.3)	66	(3.6)	**1.19 (1.07–1.33)**
*Paid work during pregnancy					
No	809	(87.2)	726	(88.6)	1
Yes	119	(12.8)	93	(11.4)	1.06 (0.94–1.21)
*Strenuous work ^e^					
No	425	(53.0)	440	(60.7)	1
Yes	377	(47.0)	285	(39.3)	**1.16 (1.05–1.27)**
*Daily workload ^f^					
≤ 8 hours	557	(69.9)	517	(71.9)	1
> 8 hours	240	(30.1)	202	(28.1)	1.05 (0.94–1.16)
Total women	2,317		1,833		

Notes: Missing data for: ^a^ 1 case; ^b^ 64 cases; ^c^ 355 cases; ^d^ 3 cases; ^e^ 220 cases; and ^f^ 231 cases. Values in bold mean are statistically significant. *Only answered by 1,747 women who had a paid job before pregnancy.

Some maternal characteristics of the obstetric history of women who had PTB varied according to ethnicity. Non-white women were more likely to have a history of ≥ 3 deliveries (PR: 1.13; 95%CI: 1.05–1.23); interpregnancy interval < 12 months (PR 1.13; 95%CI: 1.02–1.25); and pregnancy complications such as abortion (PR: 1.09; 95%CI: 1.02–1.16), PTB (PR: 1.09; 95%CI: 1.02–1.16), pPROM (PR: 1.13; 95%CI: 1.03–1.24), and low birth weight (PR: 1.08; 95%CI: 1.01–1.16) ( Table 3 ).

**Table 3:** Maternal obstetric history

Obstetric history	Non-white women		White women		Prevalence ratio (95% confidence interval)
	N	(%)	N	(%)	
Parity					
Nulliparous	1,076	(46.4)	900	(49.1)	1.0
1-2 deliveries	952	(41.1)	756	(41.2)	1.02 (0.96–1.08)
≥ 3 deliveries	289	(12.5)	177	(9.7)	**1.13 (1.05–1.23)**
Previous cesarian section ^a^					
No	1,825	(78.8)	1,422	(77.6)	1.03 (0.96–1.10)
Yes	491	(21.2)	411	(22.4)	1.0
Previous abortion					
No	1,735	(74.9)	1,437	(78.4)	1.0
Yes	582	(25.1)	396	(21.6)	**1.09 (1.02–1.16)**
*Interpregnancy interval					
< 12 months	144	(10.3)	81	(7.8)	**1.13 (1.02–1.25)**
≥ 12 months	1,255	(89.7)	959	(92.2)	1.0
Previous preterm birth ^b^					
No	1,829	(79.2)	1,497	(81.9)	1.0
Yes	491	(20.8)	331	(18.1)	**1.09 (1.02–1.16)**
Previous preterm premature rupture of membranes ^c^					
No	2,107	(91.4)	1,706	(93.5)	1.0
Yes	198	(8.6)	119	(6.5)	**1.13 (1.03–1.24)**
Previous low birth weight ^d^					
No	1,879	(82.0)	1,545	(84.7)	1.0
Yes	412	(18.0)	280	(15.3)	**1.08 (1.01–1.16)**
Total women	2,317		1,833		

Notes: Missing data for: ^a^ 1 case; ^b^ 2 cases; ^c^ 20 cases; ^d^ 34 cases. Values in bold are statistically significant. *Only answered by 2,439 women who had had previous births.

Regarding the clinical care and ANC characteristics, some unfavorable conditions were more frequent among non-white women, who were more likely to initiate ANC visits in the second or third trimesters (PR: 1.06; 95%CI: 1.09–1.23); have an inadequate number of ANC visits (PR: 1.09; 95%CI: 1.07–1.19); experience frequent physical exertion (PR: 1.15; 95%CI: 1.08–1.22); smoke in the first and second (PR: 1.16; 95%CI: 1.03–1.31) or third trimesters (PR: 1.09; 95%CI: 1.01–1.19); have anemia (PR: 1.14; 95%CI: 1.08–1.20); and gestational hypertension (PR: 1.09; 95%CI: 1.02–1.23) ( Table 4 ).

**Table 4:** Clinical and antenatal care characteristics

Clinical and antenatal care characteristics	Non-white women		White women		Prevalence ratio (95% confidence interval)
	n	(%)	n	(%)	
Antenatal care					
No	89	(3.8)	63	(3.4)	1.05 (0.92–1.20)
Yes	2,229	(96.2)	1,770	(96.6)	1.0
Onset of antenatal care ^a^					
First trimester	1,141	(61.1)	1,046	(68.9)	1.0
Second/third trimester	726	(38.9)	472	(31.1)	**1.06 (1.09–1.23)**
Adequate number of antenatal care visits ^b^					
Adequate (≥ 6)	1,386	(63.0)	1,205	(69.3)	1.0
Inadequate (< 6)	813	(37.0)	533	(30.7)	**1.13 (1.07–1.19)**
Weight gain during pregnancy ^c^					
≤ 7 kg	694	(35.9)	505	(30.9)	**1.12 (1.04–1.21)**
8-12 kg	643	(33.3)	569	(34.8)	1.03 (0.95–1.11)
> 12 kg	596	(30.8)	559	(34.2)	1.0
Physical effort ^d^					
No or rarely	1,810	(78.7)	1,523	(83.8)	1.0
Yes (often)	489	(21.3)	294	(16.2)	**1.15 (108–1.22)**
Smoking					
Never/not during pregnancy	1,955	(84.4)	1,604	(87.5)	1.0
Until the first and second trimesters	108	(4.7)	61	(3.3)	**1.16 (1.03–1.31)**
Until the third trimester	254	(10.9)	168	(9.2)	**1.09 (1.01–1.19)**
Urinary tract infection ^e^					
No	1,395	(60.9)	1,115	(61.3)	1.0
Yes	896	(39.1)	705	(38.7)	1.01 (0.95–1.06)
Vaginal bleeding ^f^					
No	1,706	(73.7)	1,361	(74.4)	1.0
Yes	608	(26.3)	467	(25.6)	1.02 (0.96–1.08)
Anemia ^g^					
No	1,271	(55.2)	1,135	(62.4)	1.0
Yes	1,030	(44.8)	685	(37.6)	**1.14 (1.08–1.20)**
*Chronic hypertension ^h^					
No	1,256	(89.5)	1,005	(89.9)	1.0
Yes	143	(10.5)	113	(10.1)	1.01 (0.89–1.13)
*Diabetes ^i^					
No	1,256	(91.2)	1,005	(89.4)	1.0
Yes	121	(8.8)	119	(10.6)	0.91 (0.79–1.03)
*Gestational hypertension ^j^					
No	1,256	(86.3)	1,005	(89.4)	1.0
Yes	199	(13.7)	119	(10.6)	**1.13 (1.02–1.23)**
*Preeclampsia/Eclampsia/Hemolysis, elevated liver enzymes, and low platelet count (HELLP) syndrome					
No	1,256	(75.2)	1,005	(76.0)	1.0
Yes	414	(24.8)	317	(24.0)	1.02 (0.95–1.09)
*Fetal growth restriction ^k^					
No	1,573	(87.4)	1350	(87.0)	1.0
Yes	227	(12.6)	202	(13.0)	0.98 (0.89–1.08)
Multiple pregnancy					
No	2,091	(90.3)	1622	(88.5)	1.0
Yes	226	(9.7)	211	(11.5)	0.92 (0.83–1.01)
Total	2,317		1833		

Notes: Missing data for: ^a^ 765 cases; ^b^ 213 cases; ^c^ 584 cases; ^d^ 561 cases; ^e^ 39 cases; ^f^ 8 cases; ^g^ 29 cases; ^h^ 475 cases; ^i^ 491 cases; ^j^ 413 cases; ^k^ 174. *Answers only available for 2,992 cases with this information included in the clinical records. **Answers only available for 3,352 cases with this information included in the clinical records. Values in bold are statistically significant.

The neonatal outcomes did not vary significantly between infants born to white and non-white women, except for the higher frequency of low birth weight (73.75% versus 69.02%; *p* = 0.0008) in infants born to non-white women, and the higher rate of seizures (4.05% versus 6.29%; *p* = 0.0085) in infants born to white women ( Table 5 ).

**Table 5:** Neonatal outcomes of preterm births according to ethnic group

Neonatal outcomes	Non-white women	White women	*p* -value
	n (%)	n (%)	
Gestational age at birth (weeks)			
< 28	178 (7.68)	130 (7.09)	0.7684
28 -34	1,023 (44.15)	812 (44.30)	
35-36	1,116 (48.17)	891 (48.61)	
Birth weight ^a^			
< 2,500 g	1,700 (73.75)	1,259 (69.02)	**0.0008**
≥ 2,500 g	605 (26.25)	565 (30.98)	
Fetal death	98 (4.23)	66 (3.60)	0.3017
Fetal malformations ^b^	233 (10.81)	191 (11.09)	0.7826
Orotracheal intubation ^c^	352 (16.15)	294 (16.92)	0.5192
*Respiratory distress	1,201 (75.77)	865 (75.81)	0.9819
*Neonatal sepsis ^d^	452 (29.50)	324 (29.37)	0.9427
*Pneumothorax ^e^	52 (3.47)	47 (4.34)	0.2569
*Seizures ^f^	63 (4.05)	71 (6.29)	**0.0085**
*Pneumonia ^g^	100 (6.45)	61 (5.42)	0.2691
Total	2,317	1,833	

Notes: Missing data for: ^a^ 21 cases; ^b^ 271 cases; ^c^ 232 cases; ^d^ 91 cases; ^e^ 145 cases; ^f^ 43 cases; ^g^ 51 cases. *Answers only available for 2,726 cases with reporting any neonatal morbidity; Values in bold are statistically significant.

## Discussion

Non-white women comprised around 56% of women who had PTB (2,317/4,150), and they had a higher proportion of unfavorable conditions related to maternal and perinatal health, such as not having a partner, age < 19 years, low level of schooling, low family income, ≥ 2 children under 5 years of age, performance of strenuous work during pregnancy, ANC visits initiated after the first trimester, inadequate number of ANC visits, smoking, anemia, and gestational hypertension. Some factors, including smoking, anemia, previous PTB, PROM or low birth weight have been recognized as remarkable risk factors for spontaneous PTB. One of the risk factors for provider-initiated PTB is gestational hypertension. 14 15 16 Despite the lack of differences between the subtype of PTB and mode of delivery, infants born to non-white women were more likely to have low birth weight, while seizures were more frequent in infants born to white women.

Various aspects of health, population and environment related to sociocultural aspects require consideration in order to understand the role of ethnicity in the complex relationship between PTB and its related outcomes. Although recent publications have not shown an association between ethnicity and spontaneous or provider-initiated PTB in the Brazilian population, 3 12 17 ethnicity has been recognized as an important factor in the prevention, diagnosis and provision of appropriate obstetric care. 18 19

A recent systematic review 19 suggested that income and the level of schooling may not be sufficient to explain why vulnerable conditions are associated with adverse maternal and perinatal outcomes such as PTB or low birth weight; however, ethnicity was strongly associated with both in that review. A study 20 comparing data from 4 population-based cohorts studies including all hospital births in 1982, 1993, 2004 and 2015 in the city of Pelotas, Southern Brazil, showed that, despite the economic advances and improvement in some reproductive health indicators in the last decades, ethnic inequalities remained stable. Poorer health indicators and conditions of vulnerability are higher among non-white women than among white women. The present study supports the evidence that ethnic inequality remains a challenge to be overcome.

In Brazil, recent maternal health programs have been launched to improve the access, coverage and quality of ANC and intrapartum care services, and some improvement in the quality of healthcare has been achieved. 21 However, ethnic inequalities in healthcare remain challenging. Historically, non-white women in Brazil have had limited access to ANC and intrapartum care services, and they have received lower quality of care. 22 23 24 According to the Birth in Brazil study, a recent national hospital-based study representative of the Brazilian population, access barriers and pilgrimage in search of delivery care were significantly more common among non-white women than among whites. In addition, blacks are at greater risk of receiving substandard care or delayed access to obstetric care during pregnancy complications. 25

Some risk factors for PTB were more frequently found in the non-white group. The distribution of risk factors may also vary between groups depending on biological and environmental characteristics. 26 27 The burden of risk factors, including cervical length, obesity, and smoking varies according to ethnic aspects. 28 On the other hand, smoking cessation programs appear to be more effective for white women. 29 Adverse cervical characteristics, such as short length and dilation, seem to occur more frequently in black than in white women. 28 In the United States, for example, preterm-related infant mortality is 54% higher among non-Hispanic blacks than among non-Hispanic whites. 30 However, we did not find any significant differences in short-term neonatal outcomes regarding ethnic groups, except for low birth weight and seizures. The EMIP study was conducted in 20 referral obstetric maternity hospitals that are part of the Brazilian Unified Health System. Universal coverage offered by the system may explain the reduced neonatal impact of unequal access to aANC in non-white women.

The current study has some limitations. Cervical length was not evaluated due to its observational nature. A multivariate analysis was not conducted, since our aim was to investigate whether the maternal characteristics of women who had PTB and its related outcomes differed according to ethnicity and not by independent risk factors for PTB per ethnic group. A strength of the study is that it is a prospective, observational, cross-sectional analysis with a large sample of women from the more populated regions in the country, with systematic and standardized data collection.

## Conclusion

No significant differences in maternal and perinatal outcomes were found between white and non-white women who had PTB in Brazil. However, several characteristics related to lower socioeconomic status and poor health were more frequently found among non-white women, showing their higher vulnerability.
